# Studies on the Interaction between Poly-Phosphane Gold(I) Complexes and Dihydrofolate Reductase: An Interplay with Nicotinamide Adenine Dinucleotide Cofactor

**DOI:** 10.3390/ijms20071802

**Published:** 2019-04-11

**Authors:** Stefania Pucciarelli, Silvia Vincenzetti, Massimo Ricciutelli, Oumarou Camille Simon, Anna Teresa Ramadori, Lorenzo Luciani, Rossana Galassi

**Affiliations:** 1 School of Biosciences and Veterinary Medicine, University of Camerino, Via Gentile III da Varano, 62032 Camerino, Italy; stefania.pucciarelli@unicam.it (S.P.); silvia.vincenzetti@unicam.it (S.V.); 2 School of Science and Technology, University of Camerino, Via Sant’Agostino, 1, 62032 Camerino, Italy; massimo.ricciutelli@unicam.it (M.R.); camilleoumarou@yahoo.fr (O.C.S.); annateresa.ramadori@unicam.it (A.T.R.); lorenzo.luciani@unicam.it (L.L.)

**Keywords:** protein–metal adducts, enzyme inhibition, DHFR, gold(I) phosphane compounds, thermodynamic data, enzymatic assays

## Abstract

A class of gold(I) phosphane complexes have been identified as inhibitors of dihydrofolate reductase (DHFR) from *E. coli*, an enzyme that catalyzes the reduction of dihydrofolate (DHF) to tetrahydrofolate (THF), using NADPH as a coenzyme. In this work, to comprehend the nature of the interaction at the basis of these inhibitory effects, the binding properties of bis- and tris-phosphane gold(I) chloride compounds in regards to DHFR have been studied by emission spectroscopy and spectrophotometric assays. The lack of cysteine and seleno-cysteine residues in the enzyme active site, the most favorable sites of attack of Au(I) moieties, makes this work noteworthy. The interaction with the gold compounds results into the quenching of the DHFR tryptophan’s emissions and in an enhancement of their intrinsic emission intensities. Moreover, a modulating action of NADPH is highlighted by means of an increase of the gold compound affinity toward the enzyme; in fact, the dissociation constants calculated for the interactions between DHFR and each gold compound in the presence of saturating NADPH were lower than the ones observed for the apo-enzyme. The fluorimetric data afforded to K_d_ values ranged from 2.22 ± 0.25 µM for (PPh_3_)_2_AuCl in the presence of NADPH to 21.4 ± 3.85 µM for ^4^L_3_AuTf in the absence of NADPH. By elucidating the energetic aspects of the binding events, we have attempted to dissect the role played by the gold phosphane/protein interactions in the inhibitory activity, resulting in an exothermic enthalpy change and a positive entropic contribution (ΔH° = −5.04 ± 0.08 kcal/mol and ΔS° = 7.34 ± 0.005 cal/mol·K).

## 1. Introduction

**Scheme 1:**
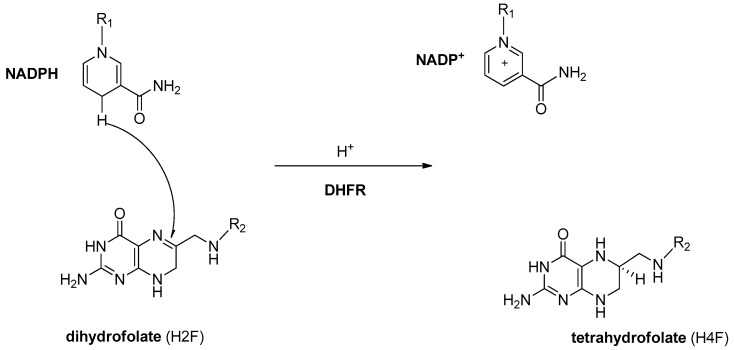
Schematic representation of the reduction reaction catalyzed by dihydrofolate reductase (DHFR).

Dihydrofolate reductase (DHFR) is a ubiquitous enzyme responsible for maintaining a pool of tetrahydrofolate (labelled as THF or H4F) in cells. THF is an active form of the vitamin folic acid and the precursor for purines, pyrimidines, and several amino acids necessary for synthesis in cells and for their growth [1]. In particular, DHFR catalyzes the reduction of 7,8-dihydrofolate (labelled as DHF or H2F) to THF by stereospecific hydride transfer from the NADPH cofactor (Nicotinamide Adenine Dinucleotide Phosphate) to the C6 atom of the pterin ring, with concomitant protonation at N5 (Scheme 1) [2]. This catalytic role makes DHFR a therapeutic target by chemical and genetic means, having the scope to hamper the proliferation and the growth of cells [3,4].

Classic antimicrobial DHFR inhibitors [5] are TMP (trimetoprin) and its analogs [4,6]; their action is mainly based on the competition with the pteridine moiety of DHF. Methotrexate is the DHFR inhibitor used most often in a clinical setting as an anticancer drug, and as an anti-inflammatory and immunosuppressive agent. Methotrexate (MTX) acts through a 10^3^ stronger bond to DHFR than to DHF itself [7,8]. The antifolate treatments may afford eventual resistance, which is one of the most important serious side effects observed, resulting in a complete loss of efficacy [9,10]. Then, the identification of new entities possessing the skill to inhibit DHFR becomes a challenging issue, as well as the study of the mechanism developing the drug resistance [11,12]. Whilst this latter aspect has been approached, the research of innovative antifolate compounds has been neglected. The design of antifolate compounds was mainly led by considering structural DHF analogies [11], and few alternative molecules have been tested as DHFR inhibitors [12]. In recent research, gold(I) azolate/phosphane compounds (where the phosphane was PPh_3_, and the azolate were some 3,5-di-substituted pyrazoles and 4,5-di-substituted imidazoles) were found to be cytotoxic in vitro against many panels of cancer cells, and two of them were found to be strongly active also in vivo on the treatment of BBLC sick syngeneic mice. They were found to possess mechanisms of action that were strongly dependent on their structure. In this regard, some years ago, our group was involved in a pioneer study where, surprisingly, these phosphane gold(I) compounds [13,14] and their relative bis-phosphane gold(I) compounds were found to inhibit DHFR from *E. coli* in the micromolar range of concentration [15]. This latter work was done with the double goal to contribute to the shared hypothesis that bioactive anticancer or antimicrobial gold(I) phosphane compounds interact with different enzymes, depending on (even mild) structural modifications [16,17], and with the aim to find new inhibitors of DHFR. Previously [15], mono- or bis- phosphane gold(I) compounds were tested as DHFR inhibitor; nevertheless, a structure activity relationship (SAR) was difficult to be discussed for mono- and bis- phosphane gold(I) compounds and some anomalies on the Ki’s values solicited a further study in depth. In order to accomplish this deepening, some gold(I) phosphane compounds were reconsidered and additional phosphane gold(I) compounds were synthesized to evaluate the nature of the DHFR/NADPH/gold compounds interactions. Exploiting the emissive properties of the tryptophan groups in DHFR, spectrofluorimetric and UV-visible spectroscopy assays [18,19,20] were performed to obtain kinetic and thermodynamic data for the DHFR/gold phosphane compounds adducts. For this purpose, PPh_3_ (L) and analogs containing the COOH in ortho (^2^L)/para (^4^L) position, or the COOCH_3_ (^4Me^L) in para position of the phosphane ligand, were used to synthesize mononuclear bis- and tris-phosphane gold(I) compounds.

## 2. Results and Discussion

### 2.1. Gold(I) Compounds Preparation

For this work, the synthesis of a selection of gold(I) poly-phosphane complexes was planned to understand some aspects of the structural/activity/relationship of these gold compounds. Specifically, (a) the change of the inhibitory activity as a function of the number of phosphane ligands around the metal center, (b) the effect of a carboxylic group in the phosphane ligand on the mechanism of interaction with the enzyme, (c) the putative role of the chloride ion in the activity of the gold complex, and (d) the binding, if indeed it occurs, between the OH of the carboxylic group and the DHFR in the interaction event. The synthesis of bis- and tris-phosphane gold(I) complexes with PPh_3_ has already been discussed in literature and it represents the inspiration for the preparation of ^4^L or ^2^L gold complexes [21]. The syntheses proceed according to the substitution of the labile (Me_2_S) by direct addition of the phosphane ligands to [AuCl(Me_2_S)] in a mole ratio 4:1, respectively; the excess of the phosphane ligand guarantees the shift of the equilibrium reaction toward the product, which is then isolated as a solid and characterized by elemental analysis, IR spectroscopy in the solid state, and ^1^H, ^31^P NMR, UV-visible, spectrofluorimetric spectroscopies, and ESI Mass Spectrometry in solution (Electro Spray Ionization Mass Spectrometry). The presence of a deactivating group, such as the carboxylic in para or ortho position of one phenyl group, confers less σ-donor skill to the phosphane ligands, labelled as ^4^L or ^2^L, yielding to quite stable gold complexes with higher coordination numbers around the gold center. The synthesis of the compound ^4^L_3_AuTfl (Tfl = triflate) was performed according to the direct reaction of the ligand, ^4^L, with the [AuTfl(Me_2_S)] in the mole ratio 4:1; this method was preferred to the direct methatesis between ^4^L_3_AuCl and AgTfl. The analysis of the ^4^L_3_AuTfl complex highlights a likely role of the chloride ion in the stability of the gold complexes in solution which was already discussed for the PPh_3_ analogs [21]: if we compare the ^31^P NMR chemical shift of ^4^L_3_AuCl and of ^4^L_3_AuTfl, we observe a shift to lower frequencies (from 42.1 to 32.6 ppm in CH_3_OD, respectively) and the appearance of a signal at 22.6 ppm for the latter (with an intensity of about 1:10 relative to the main product at 32.6 ppm). These data indicate the absence of the chloride markedly changes the electronic environment of the phosphorous center, and affords a likely dissociation to the monophosphane gold(I) compound; in fact, the ^31^P NMR chemical shift of the tris- and bis-phosphane gold(I) complexes fall quite close to each other, which are 8–10 ppm higher frequencies with respect to the chemical shift of the LAuCl derivative [15]. Finally, the formation of the methyl ester on the ligand ^4^L and of the corresponding gold(I) complex, ^4Me^L_3_AuCl, had the scope to suppress the skill of the 4-benzoic-diphenylphoshane acid ligand to act as a hydrogen bonding donor in the interaction with DHFR.

### 2.2. Inhibition Studies

The phosphane gold(I) compounds were used to prepare buffered stock solutions at pH = 7.3 at 30 °C for the inhibition tests of DHFR from E coli. In Table 1, the list of the gold(I) compounds used for the tests and the corresponding inhibition constants are reported.

**Figure 1:**
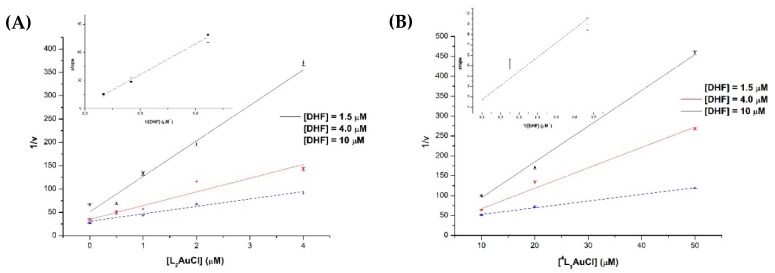
Dixon plot with the secondary plot in the inset for compound L_2_AuCl (**A**) and ^4^L_3_AuCl (**B**).

The inhibition action of the gold phosphane compounds was tested in regards to the reduction of H2F to H4F reaction, catalyzed by DHFR [2]. The inhibition tests were led by analyzing the reciprocal reaction rate upon the addition of Hepes/methanol solutions of gold compounds in different set of tests in the saturated concentration of NADPH. The tests were repeated with different amounts of dihydrofolate (DHF) (1.5 ÷ 10 µM). Typical sets of tests are reported in Figure 1.

The data were processed according to a linear regression (Equation (1)) obtaining the values reported in Table 1. The standard deviations were calculated for a minimum of three determinations.

(1) $$\frac{1}{\nu }=\frac{Km}{VmaxKi\left[S\right]}*\left[I\right]+\frac{1}{Vmax}\left(1+\frac{Km}{\left[S\right]}\right)$$

The analysis of the data shows that the most active inhibitor is the L_2_AuCl with a Ki = 0.53 µM, followed by the LAuCl and other derivatives as shown in Table 1. Modification on the phosphane ligand with a reactive group, such as the carboxylic moiety, does not improve the inhibition power, which is further worsened by its conversion to the methyl ester derivative, [(4-MeO-Ph)Ph_2_P]_3_AuCl. Notably, for the tris-phosphane gold compounds, the substitution of the chloride with the triflate ion slightly worsens the inhibition property. The change of the position of COOH from para to ortho led to an increase of the Ki values. Remarkably, by comparing the gold complexes Ki values with those of the free ligands, the central role of the gold atom on the activation of the inhibition properties is visible.

### 2.3. Fluorimetric Studies

**Figure 2:**
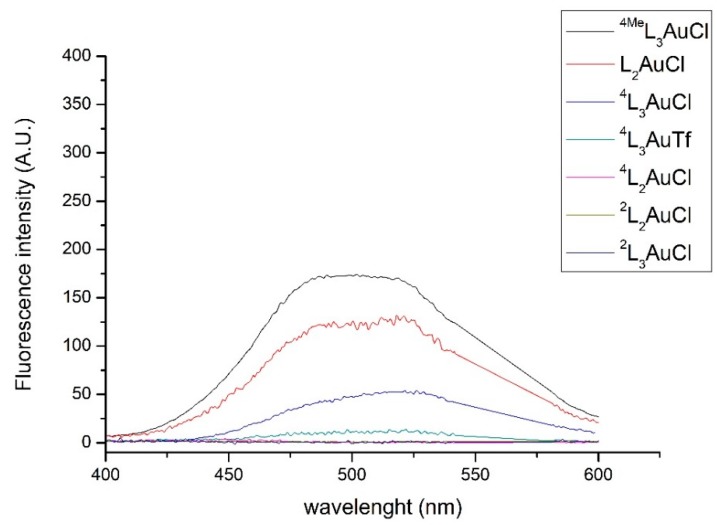
Emission spectra recorded in Hepes/methanol 22.86 µM solutions of polyphosphane gold(I) chloride compounds (λ_exc_ = 280 nm). Compounds ^4^L_2_AuCl, ^2^L_2_AuCl, and ^2^L_3_AuCl show weak intrinsic emissions and their spectra falls in the baseline intensity.

Before approaching the fluorimetric studies for the determination of the binding constants, the emissive properties of the phosphane gold(I) complexes in Hepes/methanol solutions were assessed. The fluorimetric data, both in the absence and in the presence of DHFR, are reported in Table 2. The emission spectra of compounds ^4^L_3_AuCl, ^4^L_3_AuTfl, ^4^L_2_AuCl, ^2^L_3_AuCl, ^2^L_2_AuCl, and L_2_AuCl were recorded in 20 mM Hepes/CH_3_OH solutions with concentrations of the metal complexes ranging from 4 to 50 µM. The emission’s spectra of the tested gold(I) phosphane complexes are shown in Figure 2. The most intense emissions show maxima at about 490 and 520 nm. These values are close to those observed for the (PPh_3_)_3_AuCl and can be attributed to the presence of different geometries around the gold center in solution [21]. The compound (PPh_3_)_2_AuCl, the emissive properties of which have been largely assessed in literature [21,22], is a good reference for the evaluation of the effect of the COOH group on the interaction with the enzyme. Most of the complexes showed intrinsic emissions in the yellow–green visible range by excitation at 280 nm (which corresponds to the typical absorbance of tryptophan’s in the enzyme), with a strong dependency of the intensity upon the increase of the concentration of the gold(I) compound. The emissions of these solutions are not affected by the presence of oxygen; moreover, according to the large Stokes’s shifts and the studies on halftimes reported in literature for similar (PPh_3_)_3_AuCl compounds, a likely phosphorescence decay may be attributed to these emissions. The intensity resulted in greater strength for the tris-coordinate geometry rather than for the bis-coordinate compounds [21,23]. In these PPh_3_-related compounds, the nature of the emission was explained by a charge transfer from the metal to the ligand antibonding orbitals π* ← d. Therefore, the introduction of the one COOH group slightly affects the emissive properties, and for the ester ligand derivative, ^4Me^L_3_AuCl, the most intense emission was observed [24]. 

### 2.4. Enzyme Binding Studies by Fluorimetric Assays

**Figure 3:**
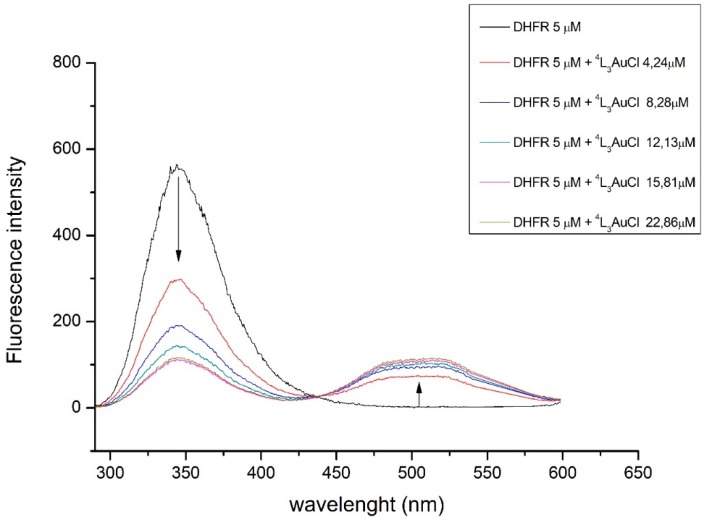
Emission spectra recorded for DHFR upon adding increasing concentration of ^4^L_3_AuCl (λ_exc_ = 280 nm). In the inset legend, the color corresponds to the curve and the respective concentrations. The spectra show the quenching of the DHFR’s tryptophan emission upon the addition of the gold compound and the concomitant increase of the emission’s intensity, attributed to the gold compound.

The Hepes/methanol solutions of the gold(I) compounds were used for the fluorimetric assays (see materials and methods section) regarding DHFR from *E. coli*. The emission’s maximum of DHFR was detected at 344 nm upon excitation at 280 nm; by adding additional amounts of the gold complex in Hepes/methanol solutions (ranging among 4 ÷ 50 µM), the quenching of this emission intensity was observed. In Figure 3, spectra showing the effect of ^4^L_3_AuCl on DHFR emission are reported. It is possible to observe the quenching of the Tryptophan’s emission and the corresponding increase of the intrinsic phosphorescence of the gold compound.

**Figure 4:**
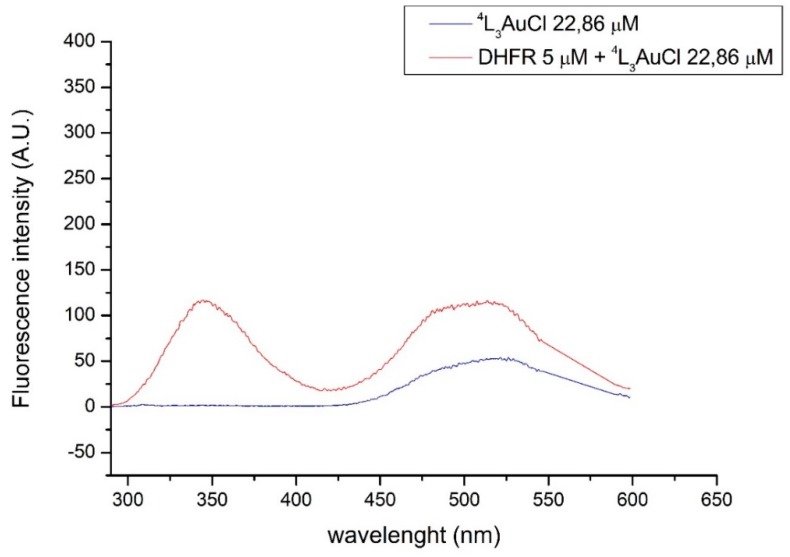
Comparison of the emission curves recorded for the intrinsic emission of ^4^L_3_AuCl in Hepes/methanol solution in the absence and in the presence of DHFR (^4^L_3_AuCl 22.86 µM in both spectra, λ_exc_ = 280 nm).

The hyperchromic effect is visible in Figure 4, where the overlaid spectra obtained for the ^4^L_3_AuCl in the presence and in the absence of DHFR are reported.

**Figure 5:**
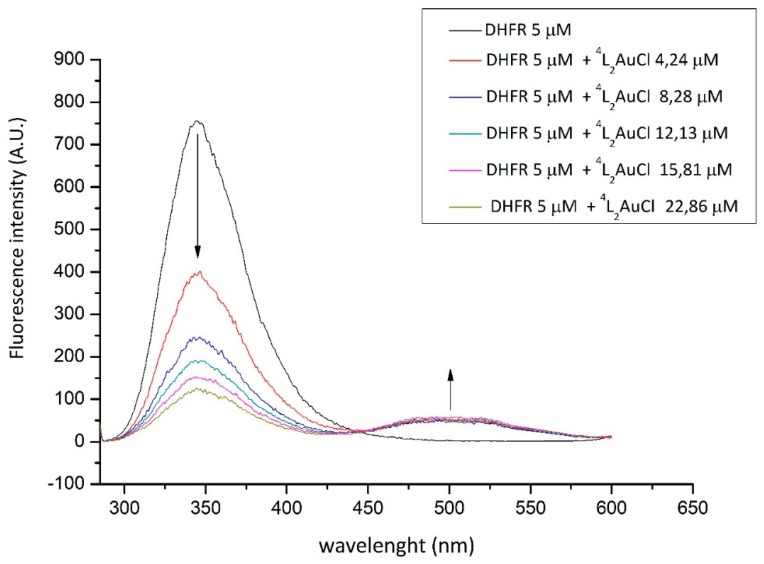
DHFR’s tryptophan emission quenching upon stepwise additions of ^4^L_2_AuCl (λ_exc_ = 280 nm). In the inset legend, the color corresponds to the curve and the respective concentrations.

**Figure 6:**
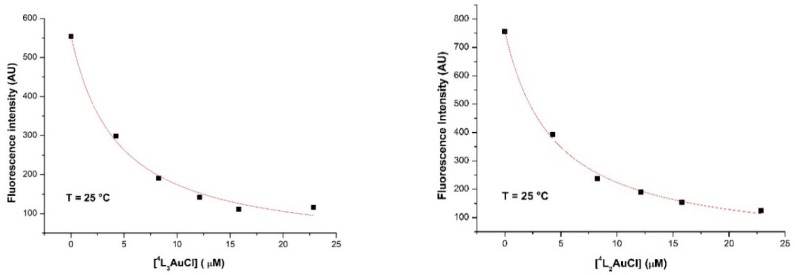
Fluorescence intensity values against added concentration of ^4^L_3_AuCl (**left**) and ^4^L_2_AuCl (**right**) at 25 °C.

The analysis of the emission’s quenching data was processed according to methods discussed in the materials and methods section. Upon treatment of DHFR with ^4^L_3_AuCl, the K_d_ value of 3.84 ± 0.29 µM was obtained. This value was compared with that obtained for ^4^L_2_AuCl (Figure 5). The emission spectrum shows a very weak emission at 511 nm, while the quenching effect is still observable. The K_d_ value for the ^4^L_2_AuCl with DHFR adduct is 4.42 ± 0.56 µM. The plots for the K_d_ analysis are reported in Figure 6.

To investigate the nature of the binding between the quencher-Au, DHFR, the free 4-benzoic-diphenyl-phosphane acid ligand, i.e., 4-COOHPh_2_P, and the benzoic acid were assayed in the same conditions. The K_d_ values are reported in Table 2 and the spectra are reported in the supplementary material section (Figure S3). These compounds, which are a structural part of the ^4^L_3_AuCl, show looser or negligible static interactions than those observed with gold compounds. These results might be explained in term of accessibility to the tryptophan of the polar benzoic acid on comparison with the lipophilic phosphane ligand, ^4^L [25]. 

**Figure 7:**
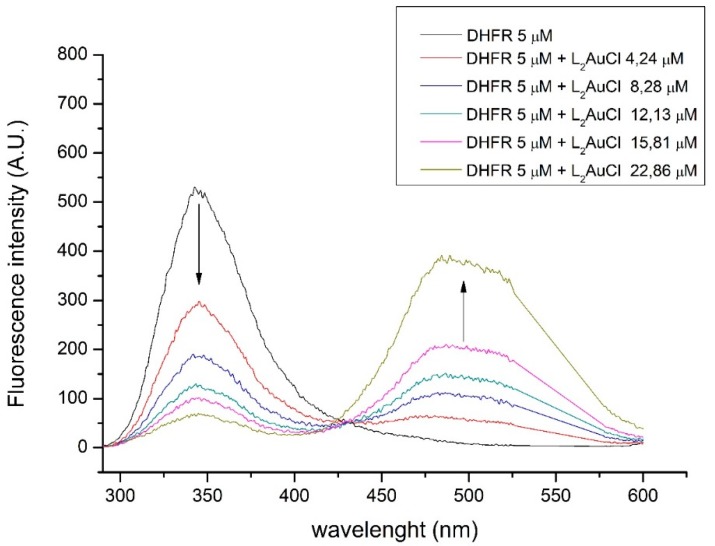
Quenching of DHFR’s tryptophan emissions according to the stepwise addition of (PPh_3_)_2_AuCl (λ_exc_ = 280 nm). In the inset legend, the color corresponds to the curve and the respective concentrations.

With the same aim, the binding of (PPh_3_)_2_AuCl (labeled as L_2_AuCl) in regard to DHFR was evaluated in terms of the binding interaction (Figure 7).

The substitution of the chloride with triflate or the esterification of the carboxylic group results in an increase of the K_d_ values, indicating less affinity of the gold complex for the enzyme than what was observed for the ^4^L_3_AuCl compound (see Table 2).

**Figure 8:**
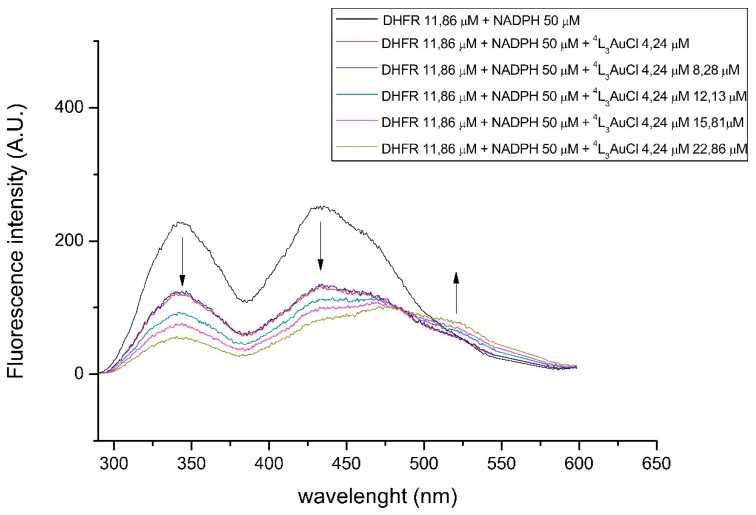
Emission spectra recorded upon the addition of Hepes/methanol solution of ^4^L_3_AuCl into the DHFR solution saturated with NADPH (λ_exc_ = 280 nm). In the inset legend, the color corresponds to the curve and the respective concentration.

After these preliminary emissive property characterizations, the fluorimetric studies were led in the presence of the enzyme DHFR and the cofactor NADPH [26]. DHFR possesses five amino-acid tryptophan residues and four tyrosine residues: both aromatic residues show intense emissive properties, and the enzyme’s tryptophan, if excited at 280 nm, exhibits an emission at 340–350 nm [18,27]. Additionally, NADPH is fluorescent, with an absorption maximum at about 340 nm and an emission maximum at about 450 nm [18]. In the presence of NADPH, the enzyme’s emission intensity is then quenched by 65%, indicating that tryptophan’s environment in the enzyme is significantly altered by the binding of cofactor. Moreover, because NADPH has a maximum of absorption at 340 nm, significant energy transfer from the excited tryptophan of the enzyme to bound NADPH occurs; as a consequence, a new emission at 420–450 nm is then observed [28]. Figure 8 reports the progressive quenching of the DHFR and NADPH emissions by stepwise adding of solution of ^4^L_3_AuCl. Moreover, a slight shift of the typical emission at 510 nm (λ_exc_ = 280 nm) is observed.

To give an insight into the emission spectra shown in Figure 8, the fluorescence spectra were also recorded for the solution of NADPH upon the addition of the gold complexes in absence of the DHFR (data reported in Table 2, rightmost column).

**Figure 9:**
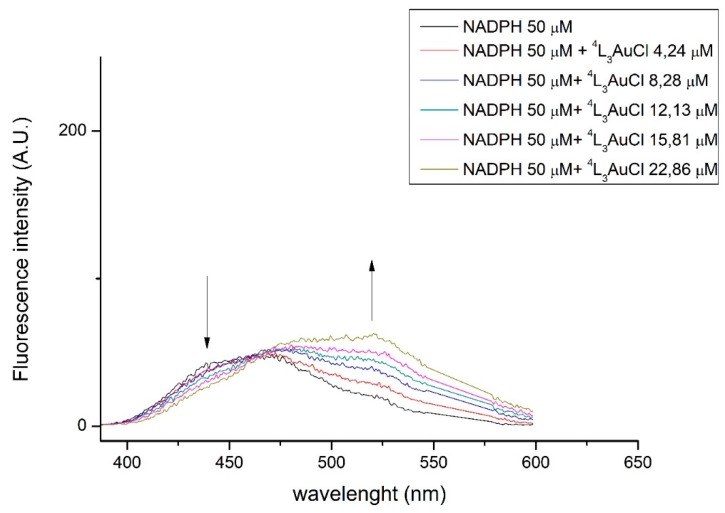
Emission spectra of 50 µM solution of NADPH upon adding an increased amount of ^4^L_3_AuCl (λ_exc_ = 280 nm).

**Figure 10:**
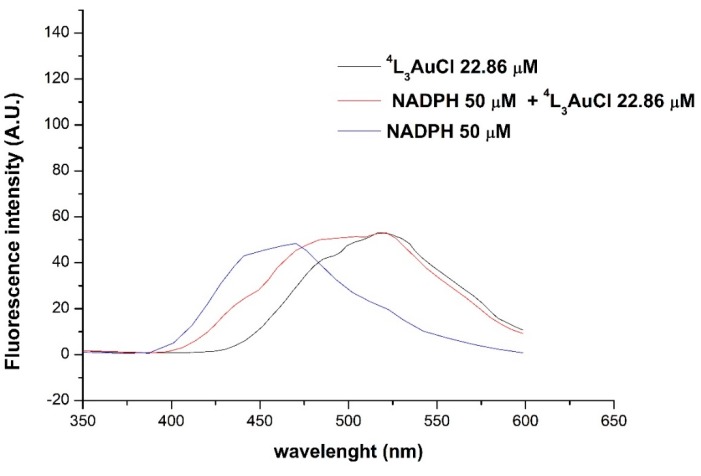
Comparison of the emission spectra recorded for NADPH 50 µM (blue line), ^4^L_3_AuCl 22.86 µM (black line), and the mixture of them (red line).

In Figure 9, the emissions recorded upon the stepwise addition of solution of ^4^L_3_AuCl to NADPH solution at 25 °C are reported (λ_exc_ = 280 nm). The spectra show a modification of the emissions attributed to NADPH, with a likely quenching either of the nicotinamide or of the adenine moieties, while the hyperchromic effect of the emission due to the gold complex is not observed. This modification of the emission maxima might be diagnostic of an interaction of the ^4^L_3_AuCl to NADPH, which is also strongly interactive with the DHFR. An interaction between NADPH and ^4^L_3_AuCl is also revealed by the emission spectrum of the mixture of the two molecules in comparison with the individual spectra, reported in Figure 10: the cumulative spectrum of NADPH and ^4^L_3_AuCl is not the resulting sum of the emission intensities of the two compounds analyzed separately.

As can be seen from Table 2, most of the K_d_ values have been obtained at different temperatures; 25, 10, and −4 °C. In general, the trend of the K_d_ values results in a lowering upon cooling, even though some discrepancies are observed mainly at −4 °C, where the solubility of the gold compounds is sometime critical. In Table 3, the fluorimetric data processed according to the vant’Hoff equation and the standard free energy equation ΔG° = ΔG° − TΔS° are reported. In all the cases, negative values of ΔG° remark the spontaneous event of the binding, with a prevalent negative enthalpy contribution, apart from the compound ^4^L_2_AuCl, for which the entropic energy prevails. Notably, this compound shows an anomaly in the trend of the Ki compared to other analogs.

### 2.5. Spectrometric Studies by ESI-MS

**Figure 11:**
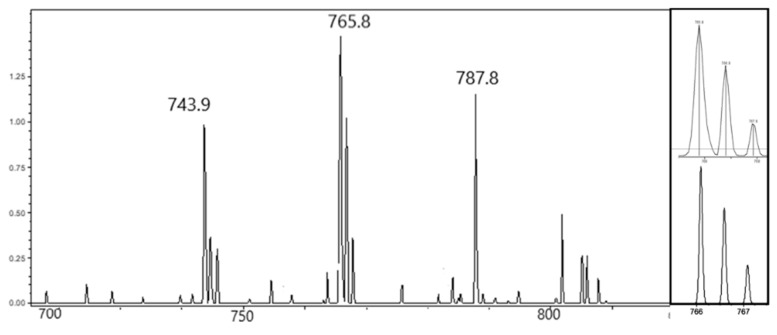
ESI-MS spectrum in the range 700–830 m/z for negative ions recorded for NADPH in ammonium acetate and ^4^L_2_AuCl upon incubation of 5 min. Magnification of the peak at 765.8 m/z for [2NADPH + ^4^L_2_Au − H_2_O]^2−^ (top, right image) and its simulation with IsoPro3.0 (bottom, right image).

Some additional studies have been led by using the ESI Mass Spectrometry [29]. In this regard, the DHFR enzyme has already been the object of several studies mainly addressed to the structural characterization [30] or to the binding of the metal to the enzyme’s fragment [31]. At first, buffered solutions of ^4^L_2_AuCl or ^4^L_3_AuCl and H2F in the presence or in the absence of NADPH were analyzed by ESI-MS to rule out the possibility of having the reduction of DHF to THF activated by gold compounds, the catalytic actions of which, in regards to many reactions, are already known [32,33]. From the analysis of the peaks and the comparison with standards of H2F and H4F, we could exclude this hypothesis. However, additional spectra were recorded on buffered solutions containing NADPH and ^4^L_2_AuCl or ^4^L_3_AuCl to analyze the interaction between the cofactor and the gold complex. In the former case, in the range 250–800 m/z, peaks due to [^4^L]^−^ (304.8 m/z), [^4^L_2_Au]^2−^ (403 m/z) and [^4^LAuCH_3_COO]^−^ (560.8 m/z) were detected; other peaks were attributed to NADPH, such as [NADPH]^2−^ at 371 m/z. The most characteristic peaks were found in the range 600–1000 m/z. In particular, in addition to the peak of [NADPH]^−^ at 743.9 m/z, a quite intense peak at 765.8 m/z might be attributed to the doubly charged ion [2NADPH + ^4^L_2_Au − H_2_O]^2−^. For this peak, a reaction between the carboxylic group and the amine function of the adenine moiety with the loss of water might be speculated. The spectrum is reported in Figure 11.

## 3. Materials and Methods

### 3.1. Syntheses

The preparation of the gold compounds was performed according to methods already published [14,15] and was based on the substitution of the labile ligand from the gold precursor [AuCl(Me_2_S)], by mean of a more coordinative ligand, such as the PPh_3_ or its modified analogs, ^4^L and ^2^L. Mole ratio with an excess of the phosphane ligand was pursued with the aim to enhance the reaction yield for tris-phosphane compounds. The solids obtained by crystallization were characterized by elemental analysis, ^1^H and ^31^P NMR, IR, and ESI Mass spectrometry. The stability of the stock solutions upon dilution was checked by recording UV-visible spectra (see Supplementary Material, Figures S1 and S2). The reactants for the ester reaction and ^4^L or ^2^L ligands were bought from Aldrich and used without any purification. Methanol or methylene chloride solvents were degassed and dried over molecular sieves.

#### 3.1.1. Synthesis of 4-(diphenylphosphanyl)benzoic acid methyl ester, ^4Me^L

The title compound was prepared according to the procedure described by Yoakim.^29^ To a 15 mL solution of 4-(diphenylphosphino)benzoic acid (398 mg; 1.30 mmol) in CH_2_Cl_2_ anhydrous, methanol (0.05 mL; 1.4 mmol) and *N*,*N*-dimethylaminopyridine (37.0 mg; 0.30 mmol) were added. A solution of N,N-dicyclohexylcarbodiimide (300 mg; 1.41 mmol) in CH_2_Cl_2_ was added over 15 min at 0 °C. The resulting pale yellow suspension was stirred for 16 h at room temperature. The suspension was filtered and dried at reduced pressure. The solid was dissolved with EtOAc (30 mL), washed successively with aqueous 10% HCl (15 mL), saturated NaHCO_3_ (15 mL), and brine (15 mL). The organic fraction was dried over MgSO_4_, filtered, and evaporated to dryness. The solid was dissolved in EtOAc and purified with flash chromatography (5% EtOAc/hexane). The product was crystallized as a white solid, with cyclohexane.

Yield: 60%.

^1^H NMR (CDCl_3,_ δ): 8.05 (d, 2H); 7.35–7.40 (m, 12 H); 3.95 (s, 3H).

^31^P NMR (CDCl_3,_ δ): −5.02 (s).

MIR (cm^−1^): 3067 (w), 2999 (w), 2952 (w), 2924 (w), 2852 (w), 2102 (w), 1943 (w), 1822 (w), 1719 (vs), 1597 (m, sh), 1584 (sh), 1561 (w), 1476 (m), 1433 (s), 1393 (m), 1278 (vs), 1181 (m), 1115 (m), 1088 (s), 1017 (s), 999 (sh), 965 (m), 857 (m), 750 (m), 743 (vs).

#### 3.1.2. Synthesis of [tris(4-benzoate-diphenylphosphanyl)-gold(I)chloride], ^4^L_3_AuCl

The solution of 4-(diphenylphosphine)benzoic acid (208.3 mg; 0.4 mmol) in CHCl_3_; was added dropwise to the solution of (dimethylsulfide)gold(I)chloride (30 mg; 0.1 mmol) in CHCl_3_; (3 mL) at 0 °C. The mixture was stirred at r. t. overnight. A pale yellow precipitate was obtained. Upon filtration, the solid was recovered and dissolved with MeOH and filtered again over a paper filter. The product was crystallized, as pale yellow solid, from a mixture of CHCl_3_; and cyclohexane.

Yield: 86%. M. p. 139–140 °C.

Analyzing the product:

^1^H-NMR (CD_3_OD, δ): 7.81 (d, 6H); 7.50 (t, 6H), 7.26 (m, 30H).

^31^P-NMR (CD_3_OD, δ): 42.09 (s).

MIR (cm^−1^): 3484 (s, br), 3054 (m, br), 1702.9 (vs), 1597.9 (s), 1560.2 (s), 1481 (m), 1434 (s), 1393 (s), 1310.9 (m), 1275 (m, sh), 1225 (vs), 1183 (s), 1122.6 (m), 1091.8 (s), 1025.9 (m), 1016.8 (s), 998.9 (m), 855 (m–w), 791.9 (m), 764 (s, sh), 742.8 (s), 689.9 (vs).

FIR (cm^−1^): 534 (w), 513.5 (m), 367.8 (w), 295.68 (w), 176.95 (m), 139 (m), 125.7 (m), 106 (w), 58.43 (m), 39.8 (m).

ESI (−) (CH_3_OH, m/z): 305.2 (100), 536.9 (40). ESI (+) (CH_3_OH, m/z): 808.9 (100) [(4-COOH-Ph_2_P)_2_Au] ^+^.

Elemental analysis for C_57_H_51_AuClO_9_P_3_, calculated%: C 56.80, H 4.26. Found C 56.86, H 4.13.

#### 3.1.3. Synthesis of [tris(4-benzoate-diphenylphosphanyl)-gold(I)triflate], ^4^L_3_AuTfl

A solution of silver trifuoromethanesulfonate (87 mg, 0.34 mmol) was dissolved in 1 mL of dry THF and then added to a solution of (dimethylsulfide)gold(I)chloride (100 mg; 0.34 mmol) in 12 mL of THF. A precipitate of AgCl was readily formed. The mixture was rapidly filtered over celite, under nitrogen flux. The clean filtrate was transferred in another flask containing the 4-(diphenylphosphino)benzoic acid in THF (2 mL). The mixture was stirred at r. t. for 3h. After evaporation to dryness, a white pale yellow compound was obtained. Then, it was washed with hexane and diethyl ether and crystallized from chloroform and hexane.

Yield: 85%. M. p. 153–154 °C.

^1^H-NMR (Acetone-d^6^, δ) 7.81 (d, J = 7.6 Hz, 6H); 7.48 (m, 6H); 7.28 (m, 30H).

^31^P-NMR (Acetone-d^6^, δ, r. t.) 32.61 (s), 22.59 (s).

MIR (cm^−1^): 3053 (m, br), 2546 (m, br), 1693 (vs), 1597.7 (m), 1560 (m), 1482 (m), 1435 (vs), 1414 (m, sh), 1395 (m), 1315 (m), 1290 (s), 1221 (vs), 1179 (m), 1157 (s,sh), 1095 (vs), 1027 (s, sh), 1017 (m), 999 (m), 926 (m, br), 854 (m), 793.3 (w), 743 (m), 690 (vs).

ESI (−) (CH_3_OH, m/z): 305.2 (100). ESI (+) (CH_3_OH, m/z): 808.9 (100) [(4-COOH-Ph_2_P)_2_Au] ^+^.

Elemental analysis for C_58_H_45_AuF_3_O_9_P_3_S + 3H_2_O, calculated%: C 56.20; H 3.69, S 2.06. Found%: C 56.07; H 3.59, S 2.53.

#### 3.1.4. Synthesis of [tris(2-benzoate-diphenylphosphanyl)-gold(I)chloride], ^2^L_3_AuCl

The solution of 2-(diphenylphosphine) benzoic acid (208.3 mg; 0.4 mmol) in CHCl_3_; was added dropwise to the solution of (dimethylsulfide)gold(I)chloride (30 mg; 0.1 mmol) in CHCl_3_; (3 mL) at 0 °C. The mixture was stirred at r.t overnight. A pale yellow precipitate was obtained. The compound was recovered upon filtration, completely dissolved with MeOH, and evaporated. The product was crystallized, as pale yellow solid, from a mixture of CHCl_3_; and cyclohexane.

Yield: 88%.

^1^H-NMR (CD_3_OD, δ): 8.51 (d, 3H); 7.73 (t, 6H), 7.45 (dt, 6H), 7.06 (m, 30H).

^31^P-NMR (CD_3_OD, δ): 47.09 (s).

IR (cm^−1^): 3054 (w), 1699 (vs), 1596 (w), 1584 (w), 1565 (w), 1471 (w), 1433 (vs), 1388 (vs), 1265 (w), 1222 (vs), 1179 (w), 1114 (s), 1091 (s), 1027, 999, 799 (s), 742 (vs), 688 (vs), 642 (vs), 522 (s), 506 (s), 446 (m), 432 (m).

ESI (−) (CH_3_OH, m/z): 305.2 (100), 536.9 (40). ESI (+) (CH_3_OH, m/z): 808.9 (100) [(2-COOH-Ph_2_P)_2_Au]^+^.

Elemental analysis for C_57_H_45_AuClO_6_P_3_, calculated%: C 61.35, H 4.06. Found C 60.98, H 4.23.

#### 3.1.5. Synthesis of [tris(4-methylbenzoate)-diphenylphosphanyl)-gold(I)chloride], ^4Me^L_3_AuCl

The solution of methyl 4-(diphenylphosphino)benzoate (64 mg; 0.2 mmol) in anhydrous CHCl_3_; (2 mL) was added dropwise to the solution of (dimethylsulfide)gold(I)chloride (15 mg; 0.05 mmol) in CHCl_3_; (3 mL) at 0 °C. The mixture was stirred at r.t. overnight, then filtered and dried at reduced pressure. The pale yellow solid was washed in hexane to remove the excess of dimethylsulfide and crystallized by CHCl_3_ solutions.

Yield: 67%.

^1^H-NMR (CDCl_3_, δ): 7.97 (d, 6H); 7.69 (d, 6H), 7.29–7.44 (m, 30H), 3.95 (s, 9H).

^31^P-NMR (CDCl_3_, δ): 30.57 (s).

MIR (cm^−1^): 3052 (w), 3008 (w), 2953 (w), 2924 (w), 2852 (w), 2103 (w, br), 1980 (w, br), 1812 (w), 1718 (vs), 1597 (m), 1559 (sh), 1560 (w), 1480 (m), 1434 (s), 1394 (m), 1273 (vs), 1186 (m), 1110 (s), 1089 (s), 1018 (s), 999 (sh), 963 (w), 952 (m), 804 (m), 743 (vs).

ESI (+) (CH_3_OH, m/z): 517 m/z (30) [(4-COOCH_3_-Ph_2_P)Au]^+^; 838 (100) [(4-COOCH_3_-Ph_2_P)_2_Au]^+^.

Elemental analysis for C_60_H_51_AuClO_6_P_3_, calculated%: C 60.39, H 4.31. Found C 60.77, H 4.76.

### 3.2. Enzyme Inhibition Studies

#### 3.2.1. Preparation of the NADPH Stock

3.2 mg of NADPH was dissolved in 1 mL of Hepes (4-(2-hydroxyethyl)-1-piperazine-1-ethanesulfonate) 50 mM buffer solution at pH = 7.3. The concentration of NADPH solution was determined by UV-Vis spectroscopy, using the buffer solution as a blank, and diluting the stock solution 1: 50 (20 mL of NADPH stock in 1 mL solution). The absorbance was recorded at 340 nm (for NADPH ε_340_ = 6220 M^−1^·cm^−1^).

#### 3.2.2. Preparation of the H2F Stock

1 mg of H2F stock was dissolved in 600 µL of HEPES buffer 50 mM and 38 µL of β-mercapto-ethanol. The KOH 0.01 M was used as the blank and to dilute the stock solution to 1:200. The absorbance was read at 283 nm (for H2F ε_283_ = 28,000 cm^−1^).

#### 3.2.3. Preparation of the DHFR Stock

The overexpressed and purified DHFR from Escherichia coli was stored at 4 °C and precipitated in a 90% (NH_4_)_2_SO_4_ solution. The dialysis of DHFR was performed in two steps with a 10 mM K_2_HPO_4_-KH_2_PO_4_ buffer, pH =7.3, the first in 3 h and the second overnight. The concentration of DHFR was determined by recording the absorbance of the stock solution at 280 nm (*E. coli* DHFR ε_280_ = 31,100 M^−1^ cm^−1^) using the potassium phosphate buffer as the blank.

#### 3.2.4. Preparation of the Gold(I) Compounds Stocks

5 mg of gold(I) compounds were dissolved in 5 mL of methanol, affording approximately 1 µM solutions. The concentration of the gold(I) compounds were calculated knowing the molar masses.

### 3.3. Inhibition Studies on DHFR

The activity of the DHFR was followed by recording the absorbance with a Shimadzu UV-2450 (UV-Vis spectrophotometer). The enzyme assays were made in a quartz cuvette by using similar experimental conditions: DHFR 3-5 nM, NADPH 60–80 μM, and H2F 1.5, 4, and 10 μM. The procedure consisted in the preparation of the following mixtures: 800–971 µL of 50 mM Hepes buffer at pH = 7.3, 20 μL of NADPH, 10 µl of DHFR, and increasing quantities of gold(I) compounds stock solutions in order to achieve higher accuracy in the measurement of the inhibitory effect. After storing the mixture for 5 min at 30 °C, 4–33 µL of H2F was added. The decreasing absorbance at 340 nm, due to the oxidation of NADPH to NADP^+^, was detected after adding the H2F, and expressed in U mL^−1^ using the equation: U·mL^−1^ = Δabs/Δt/11.8 × dilution factor, where 11.8 is the mMolar extinction coefficient when NADPH and H2F are simultaneously present in solution. The Ki values were calculated with the Dixon plot from the second plot obtained computing the slope values against the reciprocal of the H2F concentrations.

### 3.4. Fluorimetric Analysis

The fluorimetric analysis was performed using a 1 mL quartz cuvette and a Perkin–Elmer Fluorescence Spectrometer LS 55. For all the analysis we used 5–12 µM solutions of DHFR and increasing concentrations of gold(I) compounds in the 4–60 µM range. For some experiments, saturating concentrations of NADPH (50 µM) were added to the initial solutions of DHFR to observe the affinity variation of the complexes with respect to the DHFR.

To observe the intrinsic emission of the gold(I) complexes, and eventually their hyperchromic effect, emission spectra were recorded in the same range of concentration used for the quenching studies (4–60 µM).

All the emission spectra have been interpolated graphically (Origin Lab^®^ 2019) [34] in order to correct for the second order Rayleigh scattering band, centered at 560 nm.

All the spectra were recorded in the range between 250 and 600 nm using an excitation wavelength characteristic of the Tryptophan, equal to 280 nm. For most of the experiments the temperature was kept constant at 25 °C; furthermore, to analyze the thermodynamic aspects of the reactions (via the van’t Hoff equation), quenching spectra of DHFR in presence of the gold(I) complexes were recorded at −4 and 10 °C as well.

The fluorimetric data were analyzed using the binding isotherm (Equation (2)), where [Q] is the concentration of the added quenching ligand, F is the measured fluorescence, F_0_ is the starting fluorescence, Fc is the fluorescence of the fully complexed protein, K_d_ is the dissociation constant, and *n* is the Hill coefficient. Indeed, *n* is a phenomenological parameter, an interaction coefficient, such as when *n* = 1 the equation describes a complex formation with one site of interaction, when *n* = 2 binding occurs with a dimer, when 1 < *n* < 2 a cooperative interaction occurs [35].

(2) $$\frac{F0-F}{F-Fc}=\frac{{\left[Q\right]}^{n}}{{\left(Kd+\left[Q\right]\right)}^{n}}$$

### 3.5. ESI-MS Spectrometry

The instrument used was a HPLC 1100 Agilent Technologies connected to a mass spectrometer Agilent Technologies (LC/MSD Trap SL) equipped with an electrospray ion source (ESI). The analyses have been performed in FIA (flow injection analysis) mode. The mobile phase was H_2_O: MeOH 90:10 at 300 µL/min, with the addition of 0.1% formic acid. The ionization source parameters were the following: nebulizer pressure 50 psi, gas flow 10 L/min, drying gas temperature 350 °C, capillary voltage 3500 Volts, injection volume 2 µL.

The solutions of 80 µM NADPH and 100 µM ^4^L_2_AuCl have been prepared by dissolving NADPH in ammonium acetate 10 mM pH 7.3 and by adding 100 µL of a 1 mM stock solution of the gold compound dissolved in methanol, to a final volume of 1 mL.

## 4. Conclusions

In this work, a class of polyphosphane gold(I) complexes have been assayed as DHFR from *E. coli* inhibitors and a study about the mechanism of their interaction has been led. For this set of phosphane gold compounds, the strongest inhibition was detected for (PPh_3_)_2_AuCl with a value of Ki = 0.53 µM, and the same compound shows a remarkable affinity for DHFR with a thermodynamic constant for the dissociation of the adduct [DHFR:(PPh_3_)_2_AuCl] equal to about 2 µM. The presence of the COOH group in the phosphane ligand affords to compounds ^4^L_2_AuCl or ^2^L_2_AuCl, and it does not introduce a better ability to inhibit the reduction of H2F to H4F by the DHFR (Ki = 27 and 2 µM, respectively) and not even to bind the enzyme. Moreover, the conversion of the COOH into the methyl esther function in the ligand does not introduce big changes in the inhibition (K_i_ ≈ 13 µM), nor in the affinity to the DHFR (K_d_ ≈ 12.7 µM). Nevertheless, the presence of gold atom plays a central role in both inhibitory and enzyme-binding activities. The affinity of gold compounds for DHFR in general is enhanced by the presence of NADPH, which yields the interaction to be more specific and behaves as a positive allosteric modulator with respect to all the tested gold compounds. The NADPH cooperative effect on gold compounds binding by DHFR is consistent with its role in the enzyme catalytic mechanism and with the behavior observed for other DHFR inhibitors [36].

Indications about an additional action of NADPH by binding the ^4^L_2_AuCl has been also evidenced by mass spectrometry, even though the investigation was not exhaustive, the formation of an adduct between NADPH and the gold compound has been observed. In conclusion, the compounds analyzed in this paper show a reversible inhibitory effect that foresees a reversible bond, modulated by the presence of the cofactor, affected by the nature of the phosphane ligands and, unquestionably, by the presence of the gold atom. The reversible bonding is also evidenced by the hyperchromic effect on the intrinsic emission of the phosphane gold compound and by the shift of the emission maxima, due to both the stabilization of the excited state and the binding to the enzyme, respectively. The effect of the temperature on the K_d_ values (Table 3) characterizes the binding, attributing a modest exothermic contribute in spite of the entropic one. These results may be discussed by considering previous studies on the interaction of gold phosphane compounds and target enzymes, such as Thioredoxine reductase. For this latter, nanomolar IC50 values have been found [13], but for this enzyme, the formation of covalent bonds with cysteine or selenocysteine residues has been largely hypothesized [17,31]. In this work, from the kinetic inhibition and thermodynamic data, we hypothesize a reversible bond, with the enzyme DHFR having both an electrostatic and hydrophobic nature with an interplay mechanism involving the cofactor NADPH, depending on the type of the gold compound. Based on these results, we can infer that enzymes in which the catalytic site does not contain either cysteines or selenocysteines might interact with gold complexes through non-covalent interactions, endowing Ki values in the micromolar order of magnitude.

## Figures and Tables

**Table 1:** List of the compounds tested and corresponding Ki values.

Compound	Labels	Ki (µM)
[(4-COOH Ph)Ph_2_P]_3_AuCl	^4^L_3_AuCl	4.6 ± 0.5
[(4-COOH Ph)Ph_2_P]_3_AuTfl	^4^L_3_AuTfl	6.33 ± 0.7
[(4-MeO-Ph)Ph_2_P]_3_AuCl	^4Me^L_3_AuCl	12.72 ± 2.1
[(2-COOH Ph)Ph_2_P]_3_AuCl	^2^L_3_AuCl	21.26 ± 3.8
(4COOHPh-Ph_2_P)_2_AuCl	^4^L_2_AuCl	27.84 ± 12.12 *
(2COOHPh-Ph_2_P)_2_AuCl	^2^L_2_AuCl	2.066 ± 0.8 *
(PPh_3_)_2_AuCl	L_2_AuCl	0.53 ± 0.03
(4COOHPh-Ph_2_P)AuCl	^4^LAuCl	2.25 ± 0.62 *
(2COOHPh-Ph_2_P)AuCl	^2^LAuCl	1.10 ± 0.3 *
PPh_3_AuCl	LAuCl	1.214 ± 0.20 *
PPh_3_	L	6.16 ± 0.77 *
4COOHPh-Ph_2_P	^4^L	No inhibition *
2COOHPh-Ph_2_P	^2^L	No inhibition *

* For these compounds, the Ki values were obtained in a previous work [15].

**Table 2:** List of fluorescence data for the gold(I) compounds once treated with DHFR (DiHydroFolateReductase) and DHFR/NADPH (Nicotinamide Adenine Dinucleotide Phosphate), with the corresponding Kd values.

Compound	Intrinsic Emission λ_em_ (λ_exc_ = 280 nm) Hepes/methanol	Stokes Shift	Kd ± s.d. *, *T* = 25 °C (µM); *n* = Hill Coefficient *	Kd ± s.d. * (25 °C) (µM); In Presence of Saturating NADPH
^4^L_3_AuCl	520 and 485 nm	16,483 and 15,095 cm^−1^	3.84 ± 0.29; *n* = 1.73; 3.32 ± 1.28 (10 °C); 2.0 ± 0.36 (−4 °C)	5.05 ± 2.7
^4^L_3_AuTfl	510 nm weak	16,106 cm^−1^	21.4 ± 3.85; 7.36 ± 0.84 (10 °C); 8.08 ± 1.00 (−4 °C)	7.36 ± 0.84
^4Me^L_3_AuCl	517 and 500 nm	16,371 and 15,714 cm^−1^	13.4 ± 2.24; 7.63 ± 0.93 (10 °C); 21.2 ± 1.83 (−4 °C)	4.55 ± 1.04
^4^L_2_AuCl	No intrinsic emission	n. d.	4.42 ± 0.56; 3.52 ± 0.15 (10 °C); 3.06 ± 0.87 (−4 °C)	2.35 ± 0.61
^2^L_3_AuCl	No intrinsic emission	n. d.	10.47 ± 1.074; *n* = 1.23 ± 0.082; 7.12 ± 0.22 (10 °C); *n* = 1.41 ± 0.048; 5.11 ± 4.2 (−4 °C)	7.32 ± 2.46
^2^L_2_AuCl	No intrinsic emission	n. d.	10.33 ± 0.65	4.73 ± 0.68
(PPh_3_)_2_AuCl	521 and 490 nm	16,520 and 15,306 cm^−1^	5.99 ± 0.40; 2.47 ± 0.29 (10 °C); 3.83 ± 1.85 (−4 °C)	2.22 ± 0.25
4COOHPh_2_P	N.A.	n. d.	59.6 ± 5.7	N.A.
Benzoic acid	N.A.	n. d.	≈4000	N.A.

* when not specified *n* = 1; s.d. means standard deviation.

**Table 3:** Thermodynamic data for tris and bisphosphane gold complexes.

Compounds	ΔH° (kcal/mol)	ΔS° (cal/mol K)	TΔS° (kcal/mol)	ΔG° (kcal/mol)
^4^L_3_AuCl	−5.04 ± 0.08	7.34 ± 0.005	2.19 ± 0.1	−7.23 ± 0.12
^4^L_3_AuTfl	−5.32 ± 3.2	3.92 ± 8.8	1.17 ± 0.9	−4,15 ± 3.3
^4Me^L_3_AuCl	−4.35 ± n. d. *	7.69 ± n. d. *	2.29 ± n. d. *	−6.64 ± n. d. *
^2^L_3_AuCl	−3.94 ± 0.1	9.58 ± 0.08	2.86 ± 0.1	−6.8 ± 0.14
^4^L_2_AuCl	−2.02 ± 0.2	17.76 ± 0.1	5.29 ± 0.2	−7.31 ± 0.28
L_2_AuCl	−4.87 ± n. d.*	8.42 ± n. d. *	2.5 ± n. d. *	−7.37 ± n. d. *

* n. d. = not determined. Data are available only at two temperatures (10 and 25 °C) because of loss of solubility.

## Supplementary Materials

Supplementary materials can be found at https://www.mdpi.com/1422-0067/20/7/1802/s1.
