# Supplementary material for: Studies on the Interaction between Poly-Phosphane Gold(I) Complexes and Dihydrofolate Reductase: An Interplay with Nicotinamide Adenine Dinucleotide Cofactor
Source: Int J Mol Sci. 2019 Apr 11;20(7):1802. doi: 10.3390/ijms20071802 (PMC6479694; doi:10.3390/ijms20071802)
Supplement: Supplementary file 1 [file ijms-20-01802-s001.pdf]

Studies on the Interaction between Poly-Phosphane Gold(I) Complexes and Dihydrofolate Reductase: An Interplay with Nicotinamide Adenine Dinucleotide Cofactor

Stefania Pucciarelli [1], Silvia Vincenzetti [1], Massimo Ricciutelli [2], Camille Simon Oumarou [2], Annateresa Ramadori [2], Lorenzo Luciani [2] and Rossana Galassi [2,*]

1. School of Biosciences and Veterinary Medicine, University of Camerino, Via Gentile III da Varano, Camerino 62032, Italy

2. School of Science and Technology, University of Camerino, Via Sant'Agostino, 1, Camerino 62032, Italy

UV-visible spectroscopy stability tests of compounds $^4L_3$AuCl, $^4L_2$AuCl, and $^2L_2$AuCl on Hepes/methanol solution

Acquisitions of the absorptions in the range of 200–700 nm of the tested solutions were led for an hour lapse every 3 minutes in 11.85 µM concentration of $^4L_3$AuCL (figure S1), $^4L_2$AuCl, and $^2L_2$AuCl (figure S2) at 30 °C. The stability was tested in Hepes/methanol 80:20, which are the same conditions used for the inhibition tests. The spectra highlighted no overall changes in solution over the time.

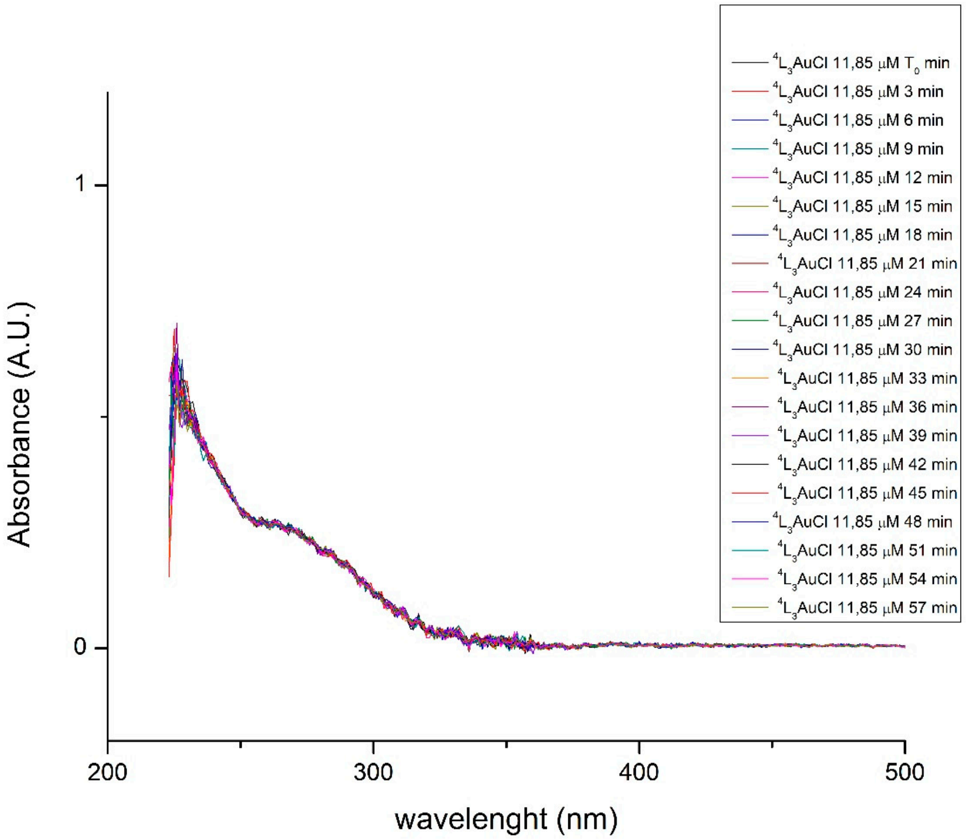

Figure S1. UV-visible spectra for 11.85 µM of $^4L_3$AuCl in hepes/methanol

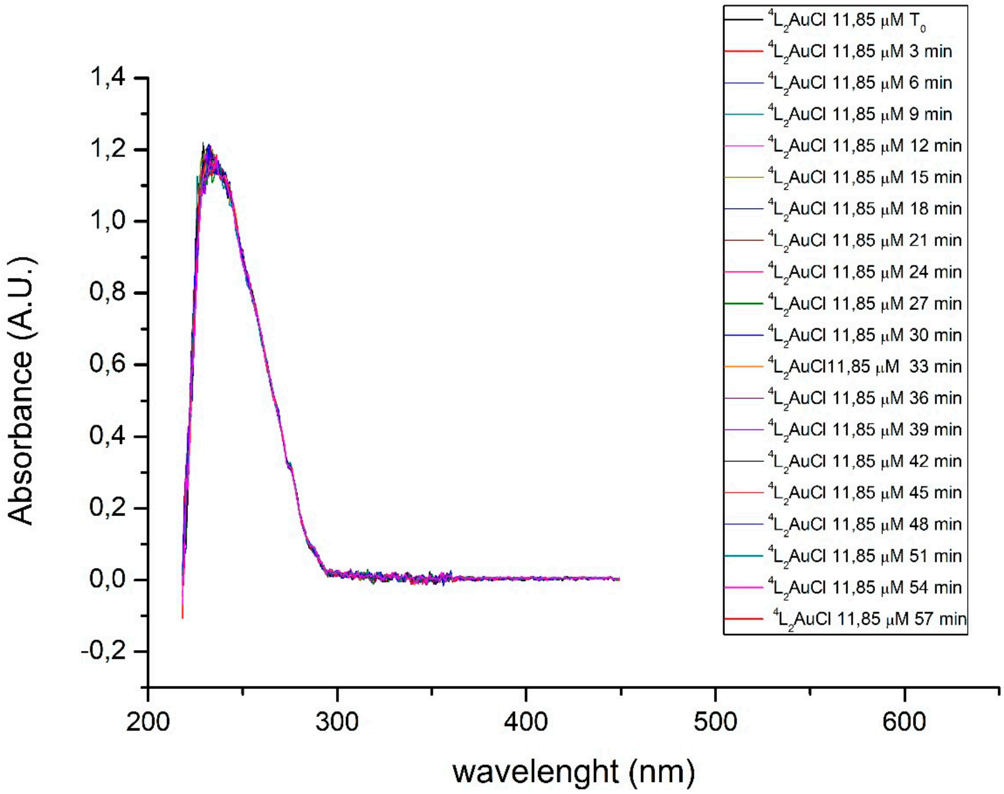

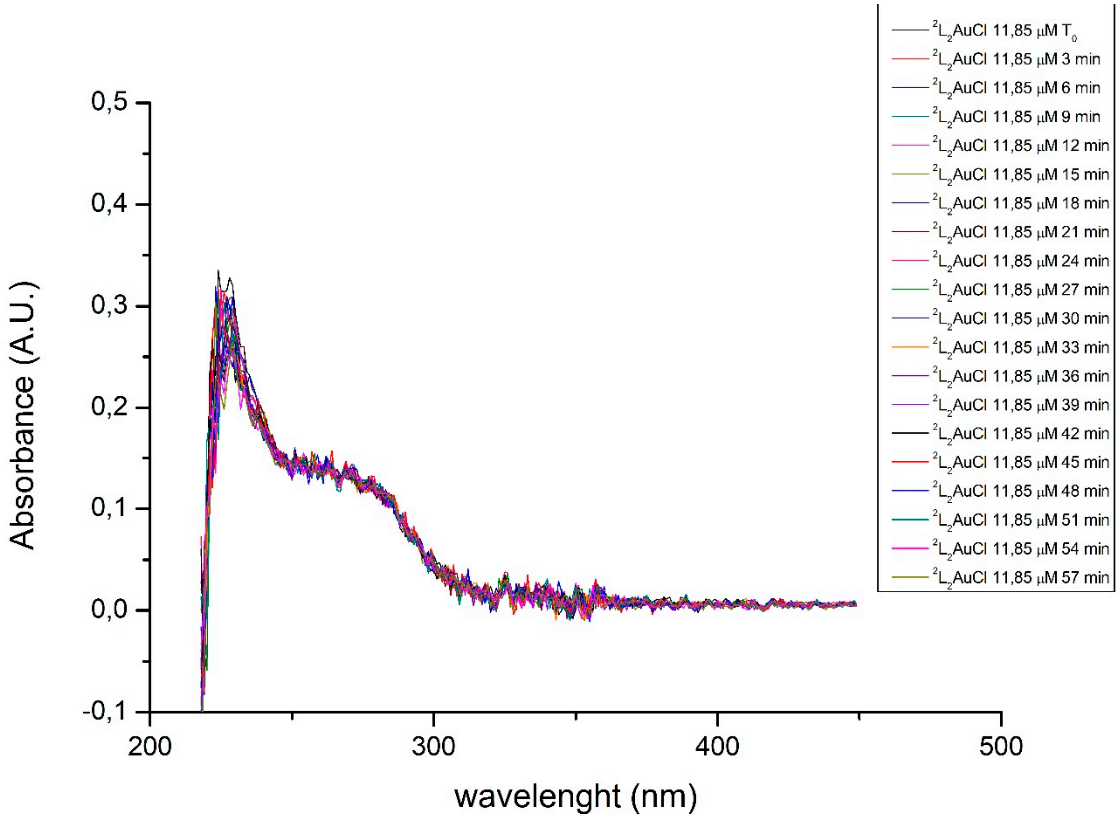

Figure S2. UV-visible spectra for 11.85 μM of $^4L_2$AuCl (above), and $^2L_2$AuCl (below) in Hepes/methanol

Emission spectra

These emission spectra were recorded upon adding ^{4}L or benzoic acid to DHFR 5μM buffered solutions.

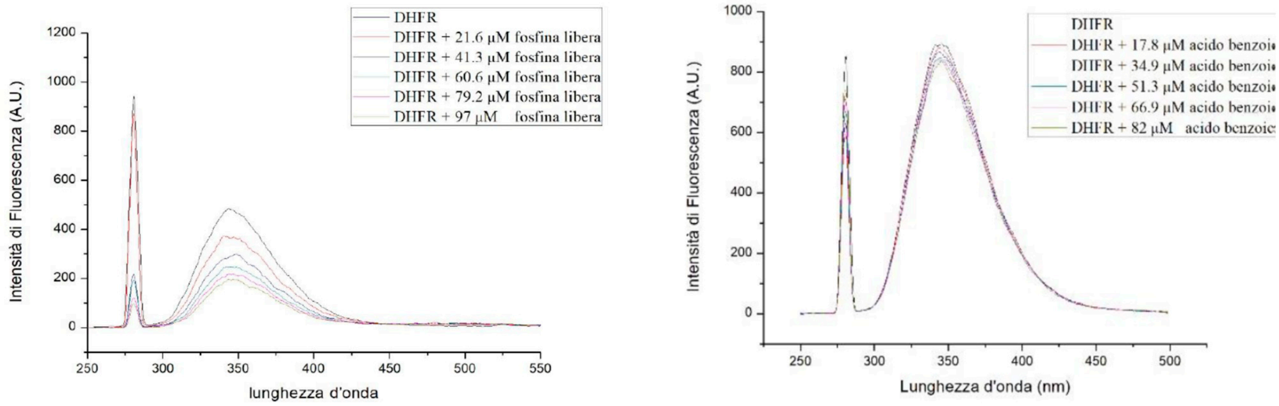

Figure S3. Quenching spectra for DHFR upon the addition of free phosphane, $4COOHPh_2P$ (left), and benzoic acid (right).
